# The post-operative loading regimen influences the regenerative potential of a biomimetic osteochondral scaffold

**DOI:** 10.1186/s12938-025-01377-6

**Published:** 2025-10-14

**Authors:** Umberto Cardinale, Alessio Pulino, Giuseppe Filardo, Marco Pes, Edoardo Fantinato, Pietro Parisse, Antonio Brunetti, Maria Antonietta Evangelisti, Lucia Manunta, Andrea Fabio Manunta

**Affiliations:** 1 AOU Sassari: Azienda Ospedaliero-Universitaria di Sassari, Sassari, Italy; 2 Faculty of Biomedical Sciences, Università della Svizzera Italiana, Lugano, Switzerland; 3 The Royal London Hospital, London, UK; 4 Istituto Officina dei Materiali Consiglio Nazionale delle Ricerche, Genova, Italy; 5 Università degli Studi di Sassari Dipartimento di Scienze Biomediche, Sassari, Italy; 6 Università degli Studi di Sassari Dipartimento di Medicina Veterinaria, Sassari, Italy

**Keywords:** ﻿Knee, Load bearing, Osteochondral scaffold, Articular cartilage, In-vivo

## Abstract

**Background:**

Articular cartilage (AC) is highly resilient and deformable. Osteochondral scaffolds have been developed to repair cartilage by mimicking the structure and function of native tissues. Our experimental model was conducted on adult sheep, commonly used as an animal model for cartilage studies, to assess the effect of early loading after osteochondral implant placement.

**Methods:**

The study was conducted on 16 adult male sheep. Cartilage defects were created and filled with an osteochondral scaffold. The sheep were divided into three groups: Group A (immobilization), Group B (partial load), and Group C (full load). Weekly clinical exams were performed, followed by micro-computed tomography (micro-CT) and atomic force microscopy (AFM) analysis on the knees, which were later collected after 6 months. The results assessed the effectiveness of partial loading compared to full immobilization or full load in terms of scaffold integration.

**Results:**

After 6 months, sheep in Group B moved without limping, whereas Groups A and C showed limited movement of the operated limb. In Group B, micro-CT analysis showed different scaffold integration and adequate osteochondral defect filling, while fibrocartilage tissue was observed in Groups A and C. Group A exhibited increased subchondral bone porosity. Group C showed increased osteochondral mineralization. AFM measurements revealed a rough surface with fiber-like structures in the cartilage area compared to the subchondral bone.

**Discussion:**

After 6 months, Group B showed better mobility recovery compared to the other groups. Micro-CT analysis revealed different scaffold integration and defect filling in Group B, while fibrocartilaginous tissue was found in Groups A and C. This study highlights the importance of controlled mechanical loading in osteochondral scaffold healing and integration.

**Conclusion:**

Our study highlighted the importance of controlled mechanical loading in osteochondral scaffold development for cartilage repair. Partial load proved favorable for scaffold healing and integration, improving mobility and reducing limping in the animal model.

## Background

The repair of osteochondral defects of the knee remains an ongoing challenge in orthopedics, aiming to restore joint function and prevent the progressive deterioration of the articular tissues. The study of osteochondral substitutes, especially in in vivo experimental settings using animal models, has opened new perspectives for joint regeneration [1, 2]. However, the long-term success of these procedures is influenced by numerous factors, including the mechanical loading imposed on the operated area [3].

Mechanical loading is known to significantly influence tissue healing dynamics and scar tissue formation [4]. However, the precise implications of loading on the response of osteochondral substitutes and the maturation of regenerated tissue remain topics of interest requiring more in-depth study. In particular, a pivotal question revolves around managing load on the operated limb immediately after an osteochondral implantation surgery [5–7]. The issue manifests in the divergence of approaches proposed by experts, creating a landscape devoid of well-defined standard protocols and clear scientific evidence.

Some surgeons suggest the necessity of immediate full load, arguing that such an approach promotes swift regaining of joint functionality and positively stimulates tissue healing processes, while others advocate for an initial partial loading to prevent excessive stress on the surgical site, thereby minimizing the risk of complications [8, 9]. In contrast, other surgeons adopt a more conservative approach by delaying weight-bearing for a significant period. This approach is based on the idea that initial protection may reduce the risk of damage to newly repaired tissues and foster more comprehensive healing [10–12]. The lack of a uniform consensus on load timing is compounded by the absence of robust scientific evidence supporting a specific approach over others [13]. While various opinions exist in the literature, the scarcity of randomized controlled clinical trials makes it challenging to draw definitive conclusions. This study seeks to explore the crucial role of postoperative loading in repairing osteochondral defects using osteochondral substitutes in an ovine model, in particular on the knee. The selection of large animal models, particularly the sheep, provides an opportunity to replicate anatomical and biomechanical conditions closer to humans, allowing for a more accurate assessment of the response of osteochondral substitutes in a physiological context.

Aim of this study is to investigate how controlled variations in mechanical loading may affect the morphology, composition, and biomechanical properties of an osteochondral substitute already used in the clinical practice with overall positive clinical results but evidence of incomplete tissue regeneration, particularly at the subchondral bone level [14].

This study is, therefore, of clinical relevance as it could provide objective evidence on how to better manage the post-operative regeneration phases of this osteochondral scaffold through optimal weight-bearing indications.

## Results

### Clinical evaluation

The post-operative clinical evaluation revealed differences in terms of lameness among the three groups under study.

In this regard, Group A cannot be considered in the context of the first month, as the limb was immobilized. After this period, lameness was evident with an associated limitation of range of motion compared to the other two groups. Moreover, in the first month, lameness in Group A was evident, and for the next 5 months, all sheep in this group never returned to apparently problem-free ambulation. Group B had a less severe lameness in the first month, followed by apparently problem-free ambulation once free housing was achieved.

### Micro-CT results

The qualitative analysis of the samples was divided into three main aspects: defect filling, subchondral porosity, and distribution of trabecular architecture. All samples were compared with micro-CT scans performed on a portion of healthy condyle, contralateral, which served as a control sample.

**Fig. 1:**
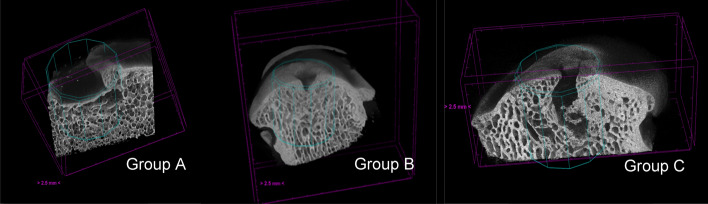
The cyan-coloured cylinder represents the scaffold position

**Fig. 2:**
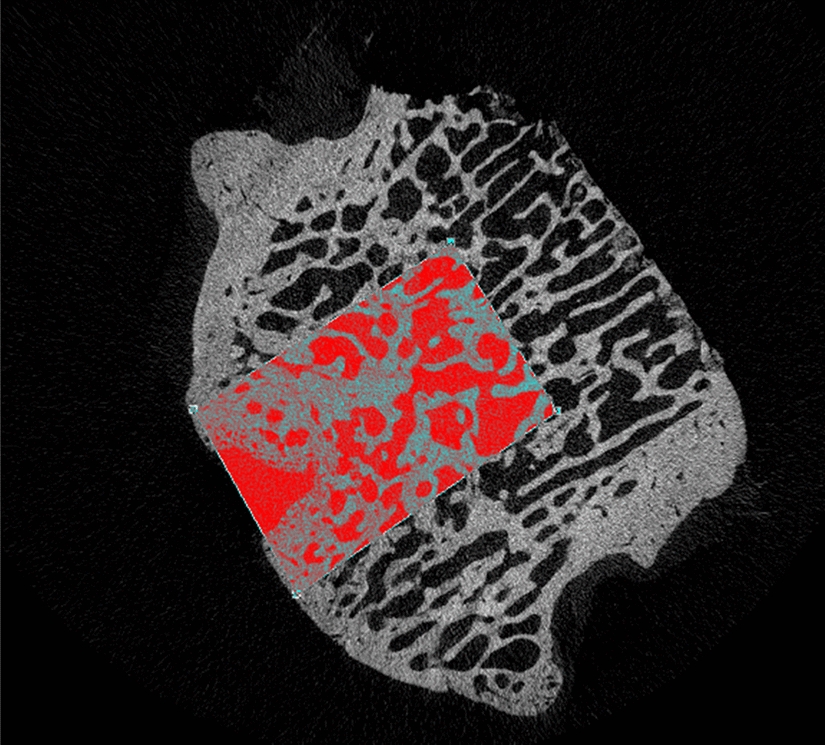
Subchondral porosity highlighted

**Fig. 3:**
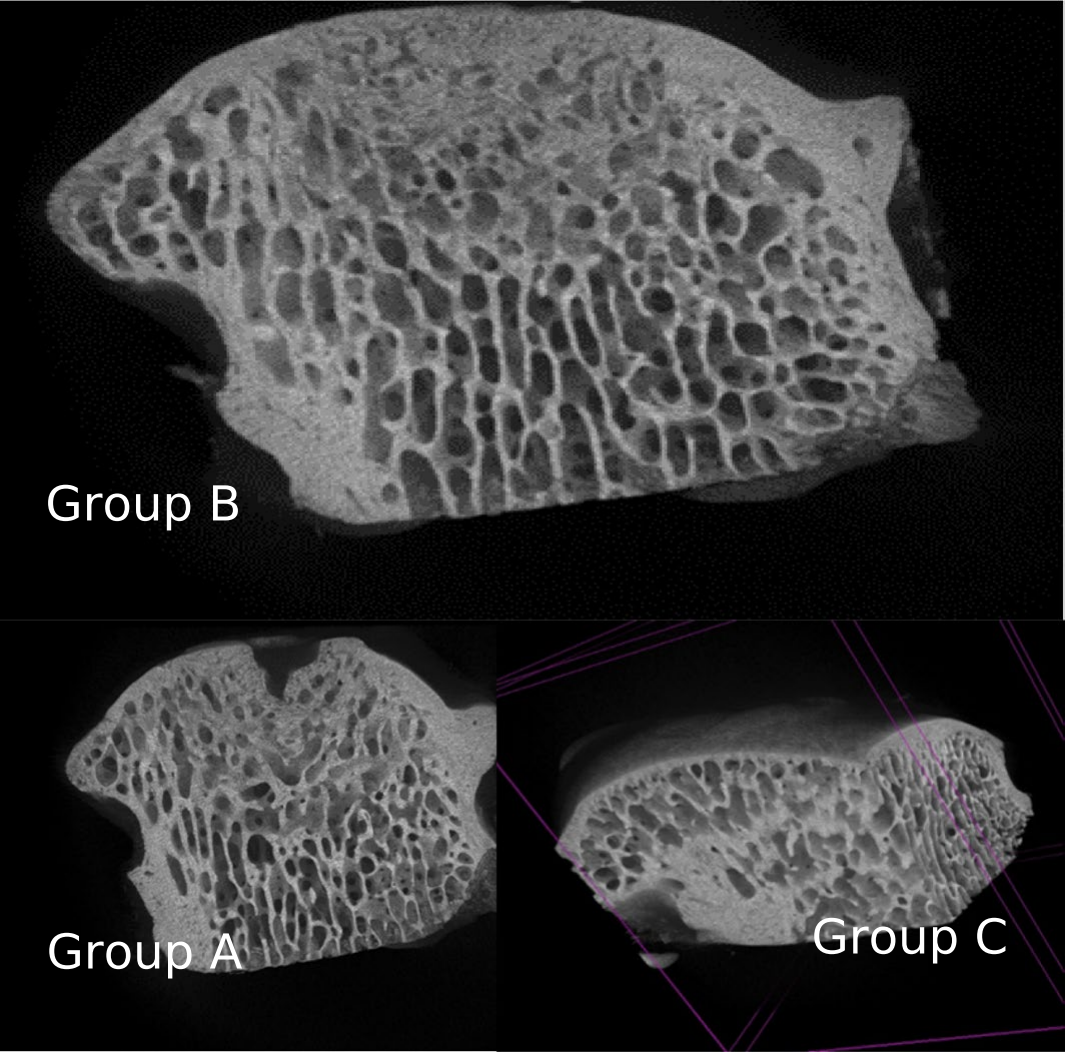
Trabecular architecture

- Defect filling: Groups A and C showed fibrocartilaginous tissue in the osteochondral defect area. In Group B, however, the scaffold appeared better integrated, with good filling of the osteochondral defect, when compared to the other two groups and the control sample (Fig. 1).
- Subchondral porosity: In Group A, a significant increase in subchondral porosity was observed. In Group C, numerous fractures of the subchondral spongy bone were highlighted. Conversely, in Group B there was improved remodeling and adaptation of the tissue layer beneath the cartilage (Fig. 2).
- Trabecular architecture: In Group A, a lower number of trabeculae was noted, whereas in Group B the trabeculae appeared to have a narrower spacing between them, indicating higher density. In Group C, increased mineralization was observed with thicker trabeculae around the osteo-chondral lesion (Fig. 3).

**Fig. 4:**
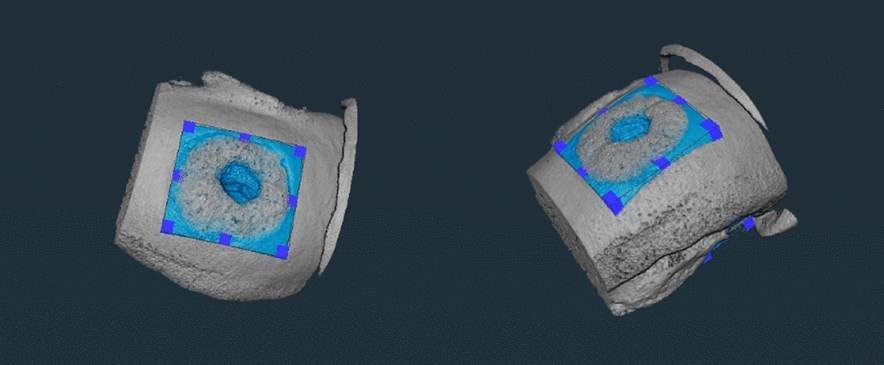
Three-dimensional visualization of sample C. In blue color the volume of interest (VOI) used for the pores analysis

To quantify the qualitative aspects described above, the Amira imaging software (Thermofisher) has been used. The approach involved extracting a section of the tissue around the implant (see Fig. 4 of sample C) and using the mathematical tools of Amira to estimate the pore diameter and volume. In Table 1, the minimum, maximum, and mean values of the diameters and volumes are reported.

**Table 1:** Minimum, maximum, and mean values of the diameters and volumes

	Diameter [pixels]			Volume [pixels]		
	Minimum	Mean	Maximum	Minimum	Mean	Maximum
Sample A	2.8	26.6	191	12	1.1 10^5^	3.68 10^7^
Sample B	3.25	24.1	64.7	18	1.1 10^4^	1.4 10^5^
Sample C	1.24	20.8	60	1	7.6 10^3^	1.1 10^5^

**Fig. 5:**
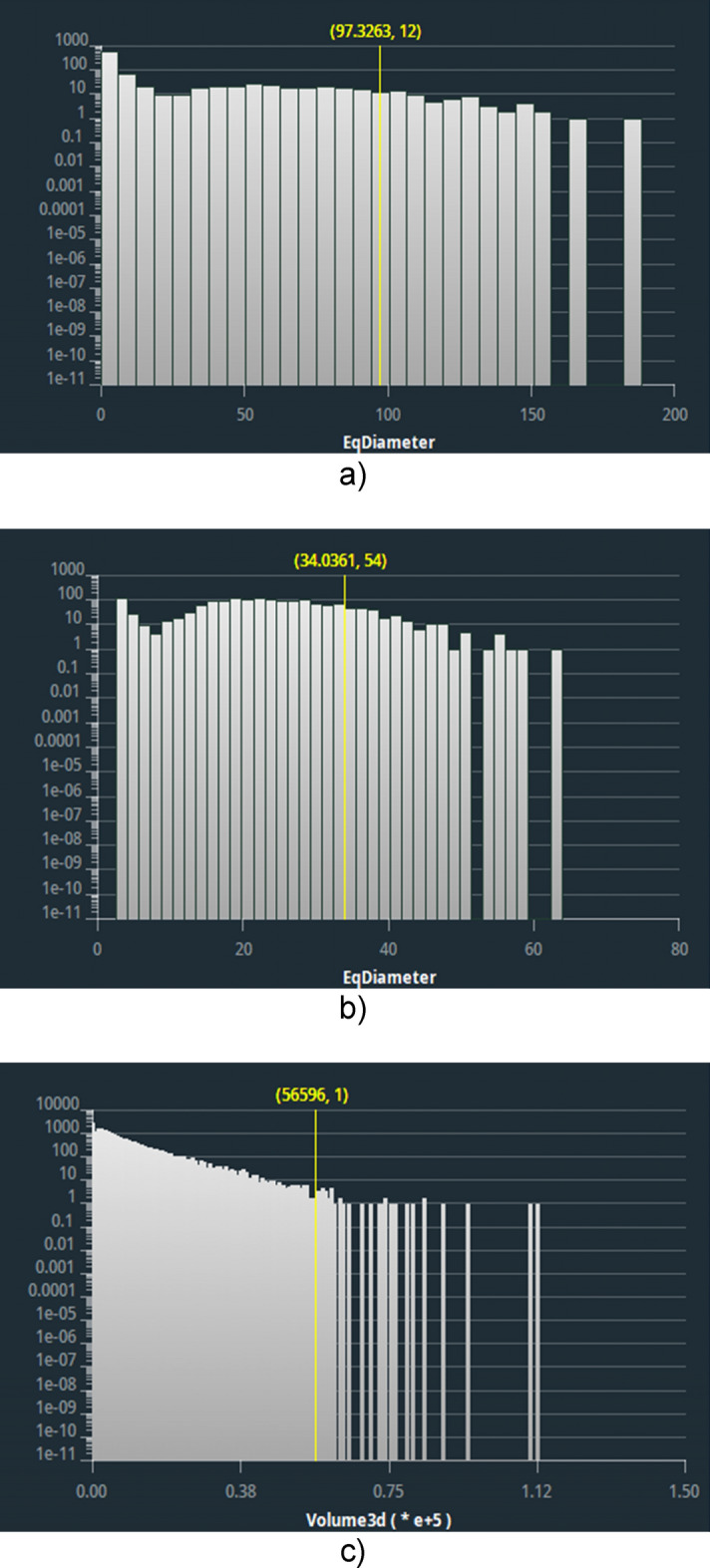
Diameters distribution histograms. **a** Sample A, **b** sample B and **c** sample C

**Fig. 6:**
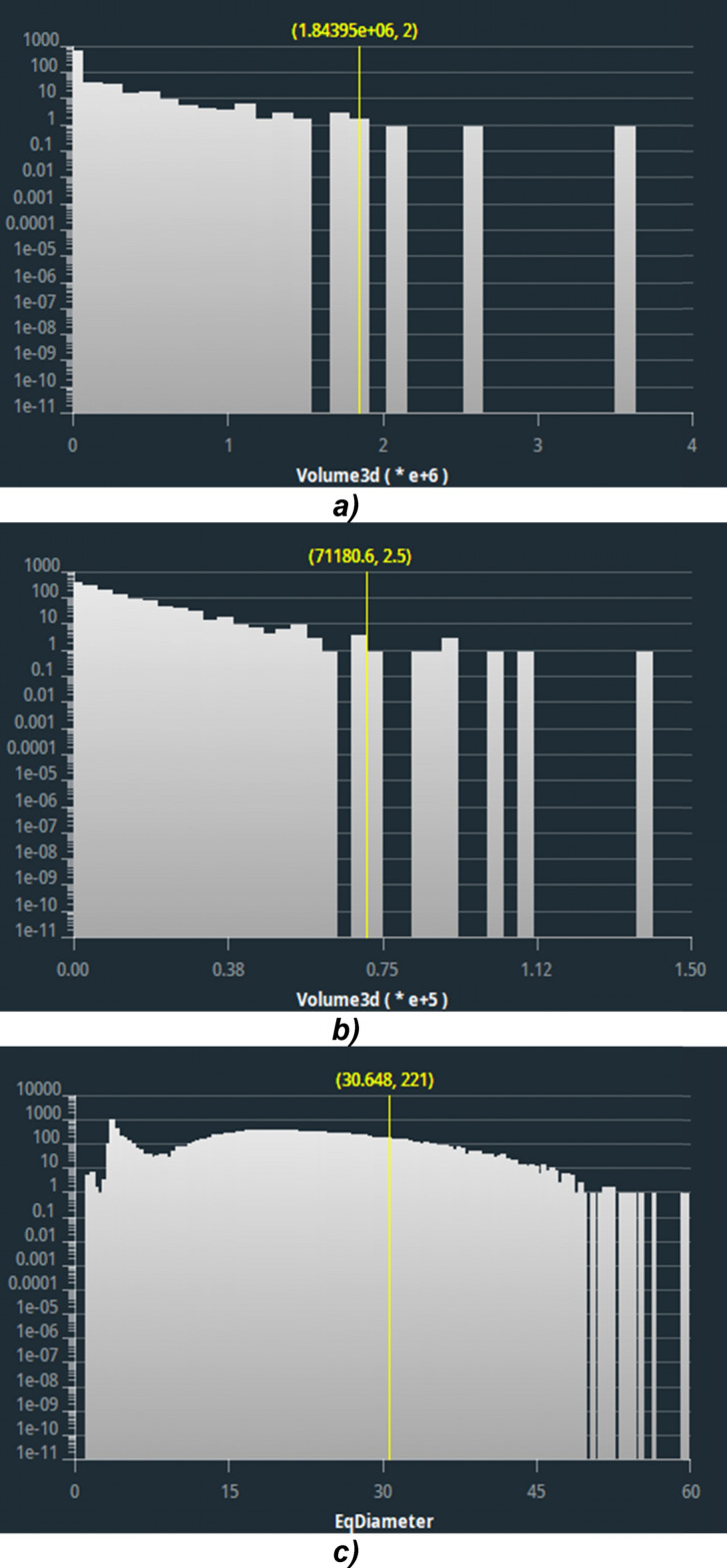
Volume sizes distribution histograms. **a** Sample A, **b** sample B and **c** sample C

Of particular interest is the distribution of the pore diameters reported in Fig. 5 and the pore volumes in Fig. 6. The diameter and volume sizes reflect the estimates obtained from the images: sample A shows a larger maximum diameter and volume size, which are due to the large void in the implant region. Sample B has a more uniform distribution of sizes with few large diameter and volume pores. Finally, sample C appears to be more compact, with a wide distribution of small diameter and volume values.

#### AFM results

We analyzed four samples (Control, A, B, C).

AYM values of different samples have been tested for normality using IgorPro Wavemetrics statistical tools (two sample tests with Runs, KS and Jarque–Bera Test), and since none of the samples resulted to be normal statistical significance was evaluated through non-parametric test (Wilcoxon Rank test).

**Fig. 7:**
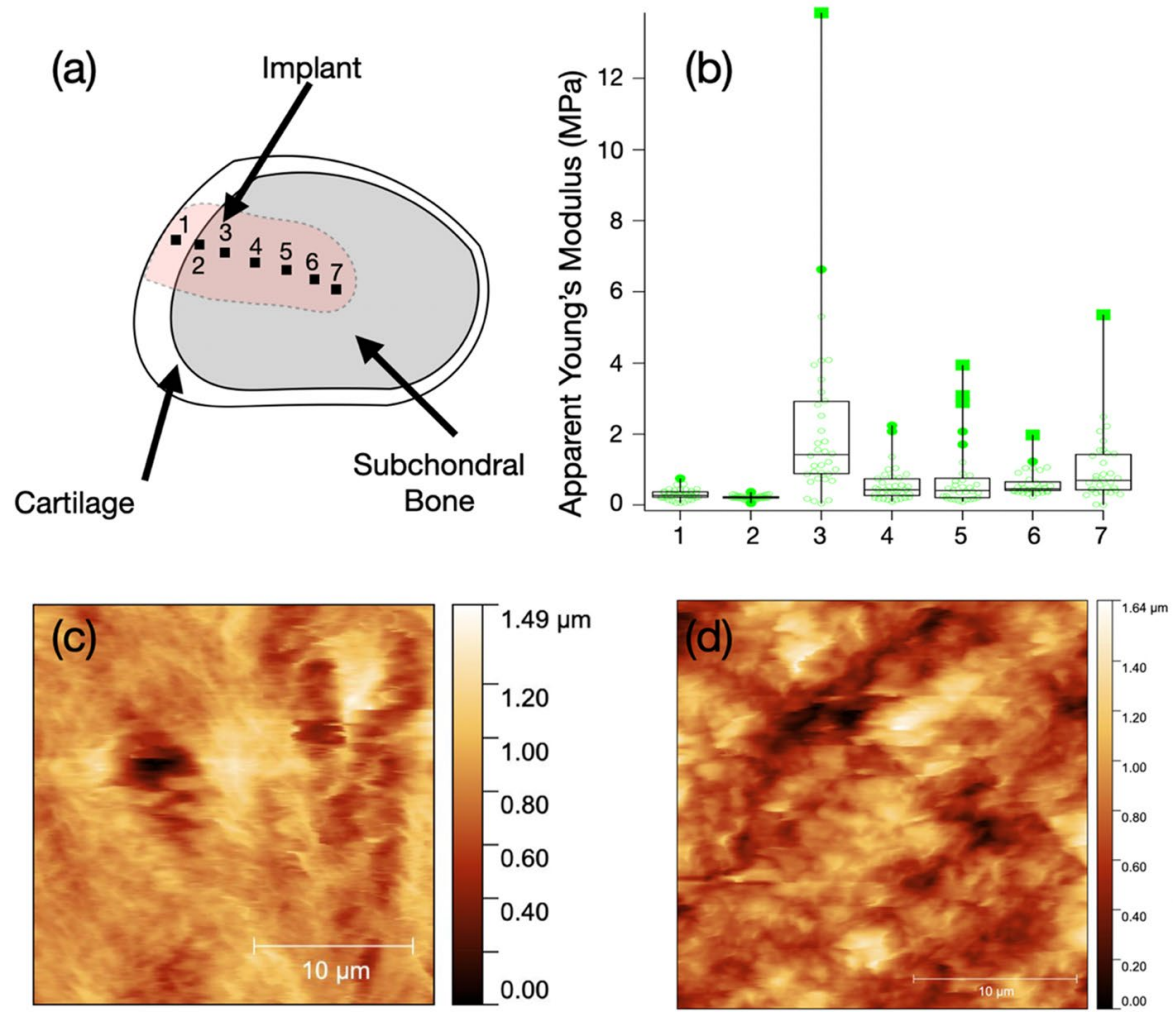
**a** Scheme of the section with the areas measured with AFM, **b** AYM values for the different points for one analyzed section; **c**, **d** Topographic AFM images of collagen (left) and bone (right)

From a morphological point of view, we did not observe appreciable differences between the different samples. The different areas showed a high roughness with fibrillar like structures more visible in the collagen area (Fig. 7c) then in the bone area (Fig. 7d).

**Fig. 8:**
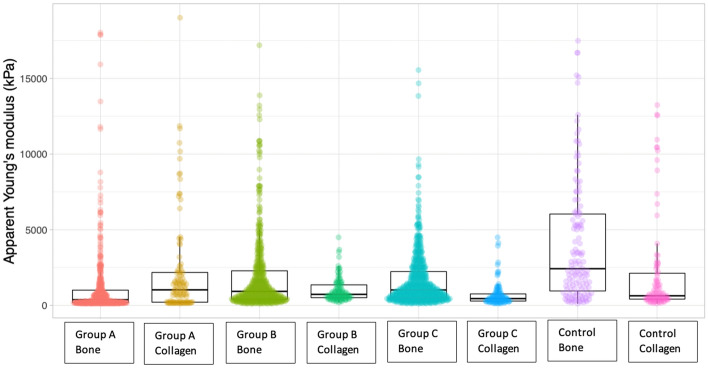
Box plot distribution of AYM of the collagen areas and bone areas for the 4 samples analyzed

Concerning the mechanical response, the analysis of the Young’s modulus for all samples revealed a high inhomogeneity of distribution of AYM across the implant, as visible in Fig. 7b. Therefore, to obtain more uniform results we decided to group together the analysis performed on the collagen areas and on the bone areas for the different samples. In Fig. 8, we report the box plot distribution of AYM for the four samples analyzed.

We observe that, apart from the Group A sample, the collagen area appears to be softer than the bone area (as expected and confirmed in the control sample). The average values for Group C and B are comparable with the one observed on the control. The bone areas values are always lower than the control sample. The Group C sample is the only one where the difference between Collagen and Bone areas is more significant (*p* < 0.0001), while in Group B the *p*-value is 0.06. The Group A sample is peculiar, due to the lower AYM of the Bone area with respect to the Collagen area. This can be due to a less efficient “calcification” of the implant that seems instead to be more consistent for the Group C sample.

## Discussion

The main finding of this study is that the different post-operative loading regimens influenced the regenerative potential and, therefore, the results of this osteochondral scaffold, which is currently applied in the clinical practice without clear evidence to guide the weight-bearing management after surgery. These results shed important light on this key aspect in the scaffold-mediated osteochondral regeneration.

Proper load or “mechanical stress” is an important factor in the success of osteochondral scaffold implantation, as it is essential for the maturation and integration of the scaffold with the surrounding tissue. Appropriate loading on the scaffold stimulates the cells within the scaffold to produce extracellular matrix and mechanical loading promotes the integration of the scaffold. However, excessive loading during the early stages of implantation can lead to undesirable effects, such as delamination of the scaffold from the surrounding tissue, inflammation, and damage to the newly formed tissue [4]. Therefore, it is important to carefully control the mechanical load on the scaffold during the initial stages of healing to ensure successful integration and long-term stability of the repaired joint.

To better understand the relationship between postoperative loading and the reparative mechanisms, advanced techniques were employed to unravel the complex dynamics involved. Two key methodologies, micro-CT and AFM, have been instrumental in providing comprehensive insights into the morphological and biomechanical aspects of the regenerated tissue. Micro-CT allows for precise three-dimensional imaging, enabling the visualization of structural changes within the osteochondral substitute under varying loading conditions. This imaging modality serves as a powerful tool to assess the spatial distribution of the substitute, offering a macroscopic perspective on how mechanical loading influences the overall architecture of the repaired region. Complementing the macroscopic view provided by micro-CT, AFM provides a high-resolution analysis of the surface properties of the regenerated tissue at the nanoscale. By probing the sample with a sharp tip, AFM captures detailed information on the topographical and mechanical properties, including stiffness. This nanomechanical characterization offers a finer understanding of how the microenvironment of the osteochondral substitute responds to different loading regimes. Overall, these evaluations method provided interesting findings demonstrating the effects of the weight-bearing regime.

The results of this study suggest that in the in vivo large animal model, group B, which underwent partial loading, had better recovery of bearing load and mobility without limping compared to the other groups at 6 months follow-up. Micro-CT data showed that the scaffold was well-integrated with good filling of the osteo-chondral defect compared to the autologous tissue and the other two groups. AFM analysis revealed an increase in surface irregularities and viscoelasticity in the superficial zone of group B. In contrast, groups A and C showed fibrocartilaginous tissue in the area of the osteo-chondral defect, with increased osteo-chondral mineralization and subchondral bone cancellous fractures detected in group A, and increased porosity and resorption found in the subchondral bone of group C. The mechanical response of the implants was also analyzed using the Young’s modulus, and the results showed high inhomogeneity of distribution of AYM across the implant. However, grouping together the analyses performed on the collagen areas and on the bone areas for the different samples we were able to confirm a less efficient calcification in group A and a more consistent one in group C.

The literature shows how exposure of the joints to chronic high intensity load eventually leads to osteoarthritis, but the absence of mechanical stimulation can cause cartilage breakdown. The mechanism which moderate load preserves cartilage integrity and function instead is still unknown [15]*.* Mechanical stimuli on cartilage produce changes within the tissue, a moderate load stimulus induces the biosynthesis of molecules to preserve the integrity of the tissue, instead mechanical overload brings to a damage of the collagen network and irreparable destruction of the tissue [16].

The timing of full weight-bearing in the rehabilitation after osteochondral restoration techniques is still unclear, the majorities of the studies suggest a progression to full weight-bearing at 6 weeks without a period of immobilization, but it depends on the technique. The protocols are still unclear regards location and size of the defect [17]. There are also several variables to consider with regard to the lesion that may have a dramatic effect on the rehabilitation process. Most importantly is the exact location of the lesion. Rehabilitation of lesions on a weightbearing surface of a femoral condyle must attempt to avoid deleterious compressive forces and require a different approach than for lesions located within the trochlea or retro surface of the patella, where excessive shear forces should be minimized. In a recent preclinical study in the large animal model of goats osteochondral lesions, the same osteochondral scaffold provided worse results at the condyle level with respect to the trochlea level, underlying the role of loading and the importance to define the most suitable weight-bearing post-operative regime [3]. The size, depth, and containment of each lesion must also be considered. Lesions that are large, deep, or have poor containment with healthy surrounding articular cartilage may require a slower rehabilitation progression than smaller, shallower, or well-contained lesions [18, 19].

Another important aspect is that the follow-up of outcomes regarding cartilage surgery techniques is not unambiguous. Modern Magnetic Resonance (MR) techniques allow a high level of resolution, after the first attempt at classification in 2006 with the MOCART system, we arrived at the MOCART 2.0 score of 2021, a semi-quantitative MR-based classification system that provides through the analysis of seven variables an estimate of the goodness of defect filling [20]. However the analysis of clinical scores through the use of Patients Reported Outcomes Measures (PROMs) remains imperative. The challenge is to correlate the proms used in knee joint surgery (KOOS, IKDC, Tegner etc.), with the imaging data [21]. Unfortunately, the radiological data often do not correlate with the clinical data reported by the patient, making interpretation of the outcomes difficult [22]. Nonetheless, the aim of the scaffold-based regenerative approaches is to restore the articular surface as close as possible to the physiological one to provide more durable results in terms of joint preservation. The strength of this study is that we were able to perform a qualitative and quantitative analysis on an in vivo biological sample that cannot be performed on humans, demonstrating the importance of a proper loading regime in the early phases after surgery.

This study certainly has some limitations. First and foremost, the animal model used does not allow for a direct translation of the results to the human model, as sheep are quadrupeds while humans are bipeds. This difference results in a distinct distribution of load, especially when discussing partial load-bearing. Humans, when necessary, can manage load with greater psychological and neuromotor awareness. Moreover, this study focused on objective tissue evaluations but lacked the MR evaluation that could be useful to translate directly the observed findings in the human setting. Nonetheless, this is the first study showing evidence on the importance of a proper weight-bearing after the implantation of a scaffold, which could serve as reference to guide post-operative indications in the clinical practice. However, the results of these studies should be interpreted with caution, and additional research is needed to confirm the safety and efficacy of these techniques in human patients.

## Conclusions

Mechanical load is a critical factor in the success of osteochondral scaffold implantation. The scaffold must be designed to withstand the mechanical loads of daily activities, and appropriate loading on the scaffold is essential for tissue maturation and integration. However, excessive loading during the early stages of healing can have negative effects on the repaired joint, emphasizing the importance of careful load control during the healing process. This study suggest that partial weight bearing provides the cartilage with a sufficient stimulus, while avoiding damages to the scaffold, and it is associated with an enhanced cartilage regeneration and subchondral bone reformation. The integration of detailed information on tissue response to different loading regimens could guide the design of more personalized and targeted therapeutic strategies, paving the way for significant advances in the clinical management of patients with osteochondral defects.

## Materials and methods

### Experimental setup

**Fig. 9:**
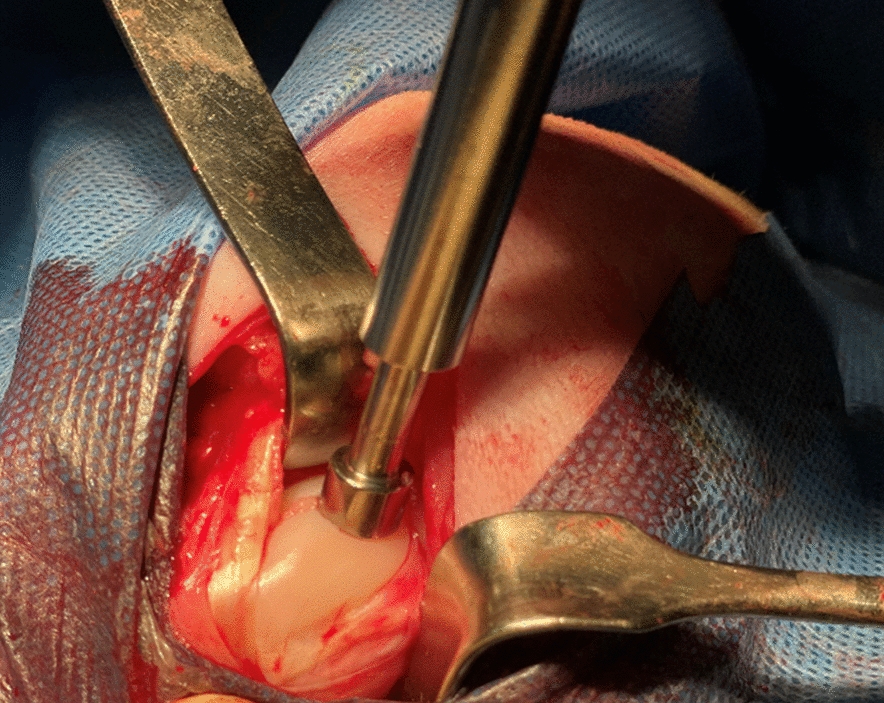
Full thickness chondral lesion

Sixteen adult male Sardinian sheep (Ovis Aries) were recruited for this study. This animal model was chosen because of its body mass is similar to humans and because it adapts quickly to the post-operative rehab [23]. Ethic committee approval was obtained for all experimental procedures prior to the beginning of the study (Ministero della Salute, Direzione generale della sanità animale e dei farmaci veterinari; Autorizzazione n. 1079/2020-PR). All procedures for animal care and treatment were performed in accordance with the Guidelines for the Care and Use of local Laboratory Animals. The animals were kept under normal conditions for 1 week before the start of the experiment to acclimatize them to the environment. After anesthesia, shaving of the entire limb and its disinfection, a parapatellar incision was performed to expose the knee joint. After the arthrotomy, we performed a standardized full thickness chondral lesion (5 × 8 mm), using a dedicated instrumentation. The defect was performed in maximum flexion of the knee, in the condyle area subjected to greater load bearing [24]. The prepared defect was filled using a properly sized osteochondral substitute (Fig. 9). The latter was a multi-layered matrix, composed by collagen and hydroxyapatite enriched with magnesium, mimicking the chondral and osteochondral tissues both in terms of chemical composition and micro- and nano-structure. More in detail, the ostechondral substitute used (Maioregen® scaffold by Fin-Ceramica Faenza S.p.A.) is a porous, nanostructured, and biomimetic material consisting of three layers, which mimic the anatomy of the osteochondral compartment. The scaffold, composed of type I collagen and hydroxyapatite substituted with magnesium (Mg-HA), aims to reproduce the morphology of cartilage and subchondral bone. The cartilage layer, forming a smooth but porous surface, is composed entirely of type I collagen; the intermediate layer (tidemark) is made up of 60% by weight collagen and 40% by weight hydroxyapatite; the lower layer, instead, is composed of 30% by weight collagen and 70% by weight hydroxyapatite to recreate a layer similar to calcified cartilage [25].

**Fig. 10:**
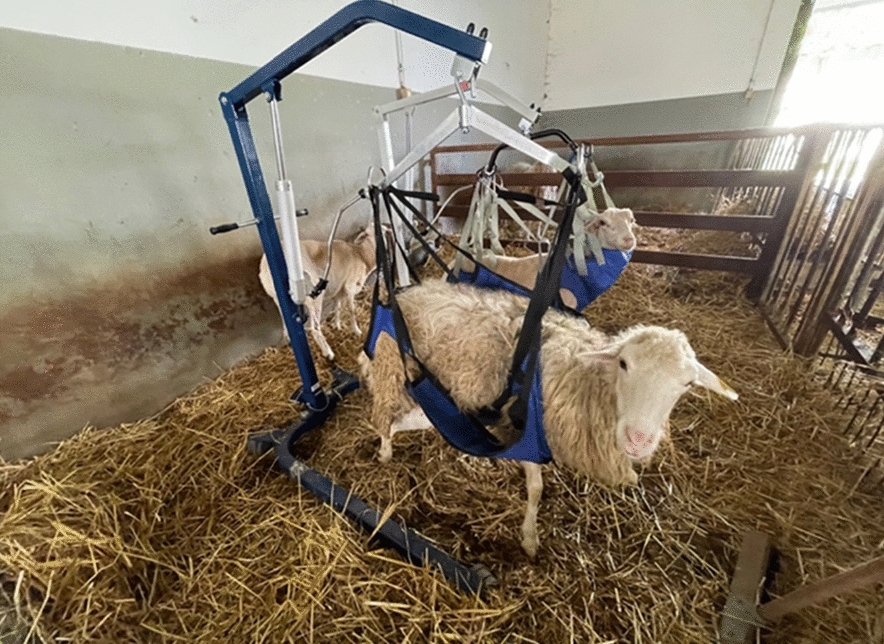
Dedicated hammock

After the operation, sheep were housed in an indoor pen with ad libitum food and water. In the post-operative period, all subjects underwent a weekly full clinical examination to assess the presence of swelling, stiffness and pain in passive movements and functional recovery of the limb. To achieve a different impact of the load bearing on the operated joint, sheep were divided into three groups: Group A included with immobilization of the operated limb by a plaster cast, applied for 1 month. Group B included sheep with partial reduction of the load on the operated limb with the aid of a dedicated hammock, used for 1 month in daytime hours (Fig. 10). The hammock allowed animals to feed and drink freely. Group C included sheep in free housing. Six months after the surgery the euthanasia of the subjects was performed. The harvested knees were immediately analyzed with micro-CT and AFM. The anatomical specimen was obtained by removing the operated condyle using dedicated surgical instruments, sized suitable for subsequent instrumental analysis. One sheep died because of infection causes about 2 weeks after surgery. Immediate post-mortem examinations determined that this death was not influenced by the scaffold used. All animals were monitored clinically for the follow-up time of the study.

### Micro-CT imaging

For the CT measurements performed here, a voltage of 100 kV and a current of 100 μA were chosen, with the beam filtered by a 0.5 mm thick aluminum layer. Before starting the CT measurements, a normalization of the camera pixels was performed. This process is known as the “flat field” correction. It consists of acquiring two images: one under the X-ray tube flux, which will be used for the CT acquisition (referred to as the "white image"), and another with the flux turned off, referred to as the "background" or "black image." The second image is subtracted from the first, and the mean pixel intensity is calculated from the resulting image. Each pixel of the final image is then divided by this mean value. The resulting matrix of values is used to correct the radiographs forming the CT measurements by dividing the pixel values of the radiographs by the corresponding correction values. In this way, the value of a pixel with higher sensitivity is reduced, while that of a pixel with lower sensitivity is increased. However, this operation can, in principle, increase the noise in the corrected image compared to an uncorrected one. To mitigate this issue, the white and black images are each formed by averaging sixteen images, which increases the signal-to-noise ratio (S/N) by a factor of four. Additionally, to further reduce the intrinsic noise of the image, each radiograph is obtained by averaging four images.

Each image was composed of 4000 × 2000 pixels, with a pixel size of 9 μm. A binning of 2 × 2 pixels was performed, which further reduces noise. However, the pixel size in the reconstructed images also depends on the magnification ratio, which is determined by the experimental setup—specifically, the ratio of the source-to-object distance to the detector-to-object distance. The closer the object is to the source and the farther it is from the detector, the greater the magnification factor. This is due to the conical shape of the X-ray emission produced by a microfocus tube. Taking into account both the binning factor and the size of the sample, the final pixel size in the reconstructed images is approximately 13.8 μm.

Each CT measurement consisted of 450 projections (with an angular step of 0.4 degrees, covering 180°). An additional correction was applied to the reconstructed images to account for beam hardening. This effect arises from the use of a polychromatic beam, such as that produced by an X-ray tube, which emits photons with energies roughly between 5 and 10 keV, depending on the filter used and the maximum voltage (in this case, 100 kV). To understand this phenomenon, consider a cylinder made of a single material and an X-ray flux incident perpendicular to its axis. The absorption of photons, as described in Eq. (1), depends on the material composition (primarily the atomic number, Z), the photon energy, and the thickness of the material traversed. Thus, low-energy photons may be completely absorbed when passing through the center of the cylinder, while only being attenuated when passing near the edges. The magnitude of this effect depends on the photon energy, resulting in the so-called beam hardening effect. This phenomenon produces circular artifacts in the image, which can be corrected, typically through interpolation between neighboring pixels. In our case, this correction is performed by the reconstruction software provided by Bruker for their Skyscan instruments.

Finally, a correction for the off-axis rotation center was applied. This correction is achieved by superimposing two profiles of the radiographs taken at 0° and 180°. From the perspective of X-ray attenuation, there is no difference between these two radiographs except that they are flipped vertically. If the left and right profiles are not perfectly superimposed, it indicates that the rotation center of the sample is not aligned with the axis of the holder. The correction is performed visually using the Bruker software by shifting one profile relative to the other. The software then applies this correction to all radiographs. It is important to note that this correction does not account for vertical tilt of the sample, so care must be taken to position the sample correctly on the holder.

### AFM imaging

The AFM analysis was focused on the evaluation of mechanical properties, and in particular of the apparent Young’s modulus, through force-distance curves. Measurements were performed on knee sections with a MFP-3D stand-alone in liquid conditions. 25 × 25 µm^2^ images and force maps were acquired in contact mode with a commercial silicon cantilever (spring constant = 0.38 N/m) in physiological solution.

Knee sections were embedded in a 35 mm petri dished filled with a two component, addition cured, platinum catalyzed, hydrophobic vinyl polysiloxane (Reprorubber® Thin Pour) to provide sufficient immobilization on the AFM stage. The hydrophobic nature of the used glue allowed to create a small pool of liquid on the exposed knee section were the AFM cantilever was introduced for the measurements. 25 × 25 µm^2^ images and force maps were acquired in contact mode with a commercial silicon cantilever (spring constant = 0.38 N/m) in physiological solution (Sodium Chloride 0.9% in 0.9% in water, BioXtra, S8776 Sigma-Aldrich).

We acquired a force map every 0.5 mm starting from the collagen towards the interior of the implant until 4–5 mm of depth (maximum depth of the implant).

Analysis of the curves has been performed with AtomicJ and IgorPro Wavemetrics softwares using a Hertzian model using as fixed parameters: radius of curvature = 100 nm, Poisson’s ratio = 0.5, invOLS = 340 nm/V, spring constant = 0.38 N/m. The analysis gave us values of Apparent Young’s modulus (AYM) in MPa. We report the box plot distribution of values for the different points across the implant for one analyzed sample (Fig. 4 panel b).

## Data Availability

No datasets were generated or analysed during the current study.

## Abbreviations

AFM: Atomic force microscopy
CT: Computer tomography
AYM: Apparent Young’s modulus
MR: Magnetic resonance
PROMs: Patient reported outcomes measures

## References

[1] Kon E, Delcogliano M, Filardo G, et al. Orderly osteochondral regeneration in a sheep model using a novel nano-composite multilayered biomaterial. J Orthop Res. 2010. 10.1002/jor.20958. 10.1002/jor.20958 19623663

[2] Filardo G, Perdisa F, Gelinsky M, et al. Novel alginate biphasic scaffold for osteochondral regeneration: an in vivo evaluation in rabbit and sheep models. J Mater Sci Mater Med. 2018. 10.1007/s10856-018-6074-0. 10.1007/s10856-018-6074-0 29804259

[3] Xu J, Vecstaudza J, Wesdorp MA, et al. Incorporating strontium enriched amorphous calcium phosphate granules in collagen/collagen-magnesium-hydroxyapatite osteochondral scaffolds improves subchondral bone repair. Mater Today Bio. 2024. 10.1016/j.mtbio.2024.100959. 10.1016/j.mtbio.2024.100959 PMC10847994 38327976

[4] Berni M, Marchiori G, Baleani M, et al. Biomechanics of the human osteochondral unit: a systematic review. Material. 2024. 10.3390/ma17071698. 10.3390/ma17071698 PMC11012636 38612211

[5] Bright P, Hambly K. A systematic review of reporting of rehabilitation in articular-cartilage-repair studies of third-generation autologous chondrocyte implantation in the knee. J Sport Rehabil. 2014. 10.1123/JSR.2013-0045. 10.1123/JSR.2013-0045 25115154

[6] Patel S, Marrone W. The evolution of rehabilitation and return to sport following cartilage surgery. Int J Sports Phys Ther. 2023. 10.26603/001c.77508. 10.26603/001c.77508 PMC10324289 37425101

[7] Della VS, Kon E, Filardo G, et al. Does intensive rehabilitation permit early return to sport without compromising the clinical outcome after arthroscopic autologous chondrocyte implantation in highly competitive athletes? Am J Sports Med. 2010. 10.1177/0363546509348490. 10.1177/0363546509348490 20051508

[8] Kane MS, Lau K, Crawford DC. Rehabilitation and postoperative management practices after osteochondral allograft transplants to the distal femur: a report from the metrics of osteochondral allografts (MOCA) Study Group 2016 Survey. Sports Health. 2017. 10.1177/1941738117717011. 10.1177/1941738117717011 PMC5665113 28719761

[9] Kosiur JR, Collins RA. Weight-bearing compared with non-weight-bearing following osteochondral autograft transfer for small defects in weight-bearing areas in the femoral articular cartilage of the knee. J Bone Jt Surg. 2014. 10.2106/JBJS.M.01041. 10.2106/JBJS.M.01041 25143504

[10] Rolf S, Kwan CK, Stoddart M, et al. Timing of postoperative weightbearing in the treatment of traumatic chondral injuries of the knee in athletes - a systematic review of current concepts in clinical practice. Asia-Pac J Sport Med Arthrosc Rehabil Technol. 2022. 10.1016/j.asmart.2022.01.001. 10.1016/j.asmart.2022.01.001 PMC8803964 35155127

[11] Tabbaa SM, Bugbee WD, Provencher M, et al. Inconsistent reporting of preauthorization medical criteria for osteochondral allograft transplantation surgery. J Bone Jt Surg. 2022;104:1841–53. 10.2106/JBJS.21.01191 35984006

[12] Mithoefer K, Hambly K, Logerstedt D, et al. Current concepts for rehabilitation and return to sport after knee articular cartilage repair in the athlete. J Orthop Sports Phys Ther. 2012;42:254–73. 22383103 10.2519/jospt.2012.3665

[13] Wondrasch B, Risberg MA, Zak L, et al. Effect of accelerated weightbearing after matrix-associated autologous chondrocyte implantation on the femoral condyle: a prospective, randomized controlled study presenting MRI-based and clinical outcomes after 5 years. Am J Sports Med. 2015. 10.1177/0363546514554910. 10.1177/0363546514554910 25378208

[14] Di Martino A, Perdisa F, Filardo G, et al. Cell-free biomimetic osteochondral scaffold for the treatment of knee lesions: clinical and imaging results at 10-year follow-up. Am J Sports Med. 2021. 10.1177/03635465211029292. 10.1177/03635465211029292 34283948

[15] Leong DJ, Li YH, Gu XI, et al. Physiological loading of joints prevents cartilage degradation through CITED2. FASEB J. 2011. 10.1096/fj.10-164277. 10.1096/fj.10-164277 PMC3005439 20826544

[16] Musumeci G. The effect of mechanical loading on articular cartilage. J Funct Morphol Kinesiol. 2016;1:154–61.

[17] Hurley ET, Davey MS, Jamal MS, et al. Return-to-play and rehabilitation protocols following cartilage restoration procedures of the knee: a systematic review. Cartilage. 2021;13:907–14. 10.1177/1947603519894733 PMC8808781 31855062

[18] Wilk KE, Macrina LC, Reinold MM. Rehabilitation following microfracture of the knee. Cartilage. 2010. 10.1177/1947603510366029. 10.1177/1947603510366029 PMC4297050 26069540

[19] Hurst JM, Steadman JR, O’Brien L, et al. Rehabilitation following microfracture for chondral injury in the knee. Clin Sports Med. 2010;29:257–65. 20226318 10.1016/j.csm.2009.12.009

[20] Stewart ZE, Joseph Simeone F, Guermazi A, et al. Postoperative imaging of cartilage: where are we in 2023? J Cartil Jt Preserv. 2023. 10.1016/j.jcjp.2023.100150.

[21] Angele P, Zellner J, Schröter S, et al. Biological reconstruction of localized full-thickness cartilage defects of the knee: a systematic review of level 1 studies with a minimum follow-up of 5 years. Cartilage. 2022. 10.1177/19476035221129571. 10.1177/19476035221129571 PMC9924981 36250517

[22] Guérin G, Pujol N. Repair of large condylar osteochondral defects of the knee by collagen scaffold minimum two-year outcomes. Orthop Traumatol Surg Res. 2020. 10.1016/j.otsr.2019.12.014. 10.1016/j.otsr.2019.12.014 32253135

[23] Barak MM, Lieberman DE, Hublin JJ. A Wolff in sheep’s clothing: trabecular bone adaptation in response to changes in joint loading orientation. Bone. 2011. 10.1016/j.bone.2011.08.020. 10.1016/j.bone.2011.08.020 21893221

[24] Taylor WR, Poepplau BM, König C, et al. The medial-lateral force distribution in the ovine stifle joint during walking. J Orthop Res. 2011. 10.1002/jor.21254. 10.1002/jor.21254 20957731

[25] Filardo G, Di Martino A, Kon E, et al. Midterm results of a combined biological and mechanical approach for the treatment of a complex knee lesion. Cartilage. 2012. 10.1177/1947603512436371. 10.1177/1947603512436371 PMC4297116 26069639

